# A phase II study evaluating the role of bortezomib in the management of relapsed acute promyelocytic leukemia treated upfront with arsenic trioxide

**DOI:** 10.1002/cam4.2883

**Published:** 2020-02-14

**Authors:** Uday Kulkarni, Saravanan Ganesan, Ansu Abu Alex, Hamenth Palani, Sachin David, Nithya Balasundaram, Arvind Venkatraman, Mani Thenmozhi, Lakshmanan Jeyaseelan, Anu Korula, Anup Devasia, Aby Abraham, Nancy Beryl Janet, Poonkuzhali Balasubramanian, Biju George, Vikram Mathews

**Affiliations:** ^1^ Department of Haematology Christian Medical College Vellore India; ^2^ Department of Biostatistics Christian Medical College Vellore India

**Keywords:** arsenic trioxide, PML mutations, proteasome inhibitor, relapsed acute promyelocytic leukemia

## Abstract

The standard‐of‐care for patients with acute promyelocytic leukemia (APL) relapsing after upfront arsenic trioxide (ATO) therapy is not defined. The present study was undertaken to evaluate the safety of addition of bortezomib to ATO in the treatment of relapsed APL based on our previously reported preclinical data demonstrating synergy between these agents. This was an open label, nonrandomized, phase II, single‐center study. We enrolled 22 consecutive patients with relapsed APL. The median age was 26.5 years (interquartile range 17.5 to 41.5). The median time from initial diagnosis to relapse was 23.1 months (interquartile range 15.6 to 43.8). All patients achieved hematological remission at a median time of 45 days (range 40‐63). Nineteen patients were in molecular remission at the end of induction. Grade 3 adverse events occurred in eight instances with one patient requiring discontinuation of therapy for grade 3 neuropathy. Twelve (54.5%) patients underwent autologous transplantation (auto‐SCT) in molecular remission while the rest opted for maintenance therapy. The median follow‐up was 48 months (range 28‐56.3). Of the patients undergoing auto‐SCT, all except one was alive and relapse free at last follow‐up. Of the patients who opted for maintenance therapy, three developed a second relapse. For treatment of APL relapsing after upfront ATO therapy, addition of bortezomib to a standard ATO‐based salvage regimen is safe and effective. This trial was registered at http://www.clinicaltrials.gov as NCT01950611.

## Graphical abstract

The standard‐of‐care for patients with acute promyelocytic leukemia (APL) relapsing after upfront arsenic trioxide (ATO) therapy is not defined. In this phase II study we demonstrate the safety of adding a proteasome inhibitor with ATO in the management of relapsed APL treated with upfront ATO. We also demonstrate the efficacy of this combination and the potential for its utilization in this setting.

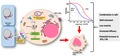

## 1 INTRODUCTION

Acute promyelocytic leukemia (APL) is a subtype of acute myeloid leukemia (AML) with distinct molecular and clinical features and characterized by the presence of a reciprocal translocation involving a portion of the retinoic acid receptor alpha gene (RARα) on chromosome 17 and a variable portion of a partner gene, which in 95% of cases is the PML gene on chromosome 15; t(15;17)(q22;q21).^1^ This reciprocal translocation results in the production of a fusion onco‐protein PML‐RARA which is central to initiating and maintaining this subtype of leukemia.^2^ Significant advances in the management of APL have resulted in this subset of leukemia having the highest cure rates.^3^ Additionally, these advances have been brought about by the use of differentiating agents and a nonmyelotoxic approach.^4^, ^5^, ^6^ The combination of all‐trans retinoic acid (ATRA) and arsenic trioxide (ATO), a nonmyelotoxic approach, has been established as the standard of care based on a phase III randomized control trial for patients with low and intermediate risk APL that accounts for more than two‐thirds of all APL patients.^4^, ^5^, ^7^ The current understanding of the mechanism of action of ATO and its efficacy in clearing the leukemia initiating compartment is based on its ability to clear the PML‐RARA onco‐protein which in turn is dependent on an intact functional proteasome and proteasomal degradation of this onco‐protein.^8^, ^9^ For high‐risk disease, most protocols that are currently used would add an anthracycline or a combination chemotherapy schedule in induction and consolidation in addition to ATRA with or without ATO.^5^, ^10^, ^11^, ^12^, ^13^, ^14^ Despite these advances disease relapse remains a challenge especially in high‐risk APL and about 10%‐20% patients with APL treated upfront with ATRA and chemotherapy will relapse.^15^ The current standard of care for patients who do relapse is to reinduce molecular remission with ATO either alone or in combination with other agents, such as gemtuzumab which is effective in this setting, and to follow this up with an autologous stem cell transplantation (auto‐SCT) in second molecular remission.^16^ It is anticipated that with this approach one could potentially cure 60%‐70% of these patients.^16^, ^17^ However, it must be noted and recognized that the available data with management of relapsed APL are from the era where ATO was not used as upfront therapy. With ATO increasingly being used as part of upfront therapy the optimal approach to management of patients who have already been exposed to ATO has not been described. Available data on response and survival outcomes in patients who had received ATO as part of upfront therapy suggests that resistance to ATO can occur resulting in inferior response and inferior survival than seen in patients who had not received ATO as part of upfront therapy.^18^, ^19^ This has been attributed to mutations in the oncogenic PML‐RARA gene following treatment with ATO which have been reported to occur in one‐third of patients with relapsed APL and the clinical outcomes in this subset with mutations is poor.^19^

We had previously reported a comprehensive evaluation comparing newly diagnosed and relapsed APL patients who had received upfront treatment with ATO, we noted that reinduction with ATO at relapse is effective; however, in spite of achieving molecular remission in the majority of patients the risk of subsequent relapse is high especially in the absence of consolidation with autologous transplantation.^16^, ^17^ We also demonstrated that there was significant microenvironment‐mediated drug resistance (EM‐DR) to ATO, which is predominantly mediated by the upregulation of the NF‐ κB pathway and is more prominent in relapsed APL.^20^ This EM‐DR to ATO could be overcome by the use of proteasome inhibitors in‐vitro and in a preclinical mouse model.^21^ In contrast to expectations, based on the known mechanism of action of ATO, we were able to demonstrate that malignant promyelocytes were exquisitely sensitive to bortezomib a known proteasome inhibitor and demonstrated significant synergy on combining it with ATO both in an in‐vitro system and in a mouse model of APL.^21^ We further clarified the mechanism of PML‐RARα onco‐protein degradation on combining these two agents by a proteasome‐independent pathway.^21^ Based on our promising preclinical data we hypothesized that the combination of ATO and bortezomib would be clinically effective at APL relapse and could potentially obviate the need of an auto‐SCT.

## 2 MATERIALS AND METHODS

### 2.1 Study design and patient eligibility

This was an open label, nonrandomized, phase II, single‐center interventional trial with single group assignment to bortezomib in addition to conventional therapy. The trial was approved by the institutional ethics committee (IRB 8225 27/02/13) and was registered in the public domain (Clinical Trials.gov: NCT01950611 and CTRI No: REF/2014/08/007490). Any patient with hematological relapse of PML‐RARA positive APL was eligible to be enrolled in this study. The detailed inclusion and exclusion criteria are provided in the supplementary methods. Some of the key exclusion criteria were intracranial bleeding at presentation, Eastern Co‐operative Oncology Group (ECOG) performance score ≥ 3, severe uncontrolled infection, cardiac dysfunction and secondary APL. All patients were enrolled after getting written and informed IRB approved consent or assent form as appropriate.

### 2.2 Study protocol

**Figure 1:**
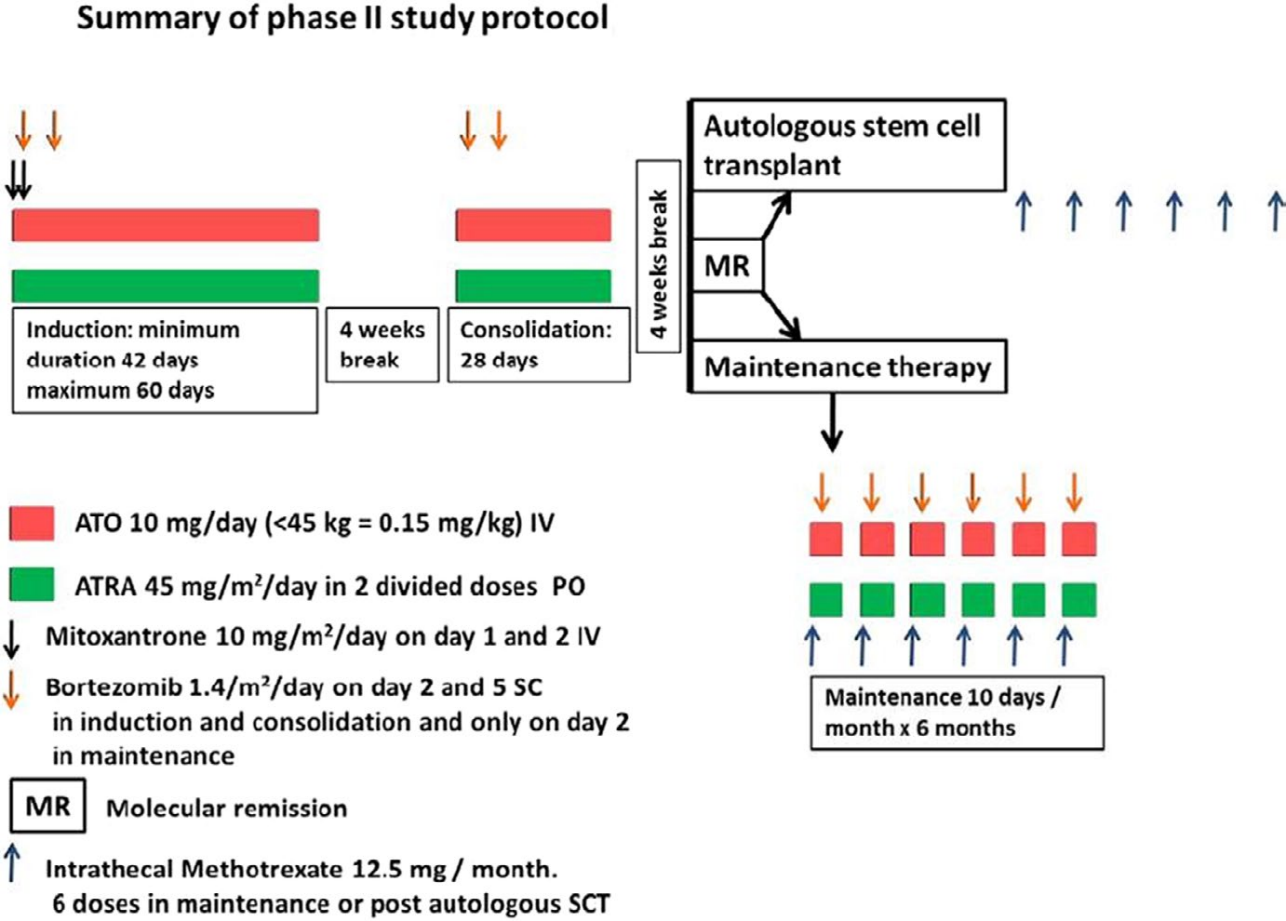
Overview of treatment schedule. ATO: Arsenic trioxide, ATRA: All trans‐retinoic acid (ATRA). During induction therapy, enrolled patients received ATO and ATRA for a minimum duration of 42 d and a maximum duration of 60 d. Mitoxantrone for 2 d was given on the first 2 d of induction. Patients also received two doses of bortezomib 1.4 mg/m^2^/dose SC on days 2 and 5. Consolidation therapy consisted of ATO and ATRA for 4 wks along with 2 doses of bortezomib. Patients who were in molecular remission post consolidation were offered SCT. Patients in molecular remission who due to various reasons could not proceed to a SCT were offered maintenance therapy with ATO and ATRA given for 10 d in a month for 6 mo. These patients also received one dose of bortezomib during each month of maintenance. Post auto‐SCT or with each maintenance cycle, intrathecal methotrexate was administered once a month for 6 mo

The treatment schema is summarized in Figure 1. Briefly, during induction therapy, enrolled patients received ATO 10 mg/day IV (or 0.15 mg/kg for patients weighing less than 45 kg) and ATRA 45 mg/m^2^/day PO for a minimum duration of 42 days and a maximum duration of 60 days. Mitoxantrone 10mg/m^2^/day IV for 2 days was given on the first 2 days of induction. Additionally, patients received two doses of bortezomib 1.4 mg/m^2^/dose SC on days 2 and 5. Patients with central nervous system (CNS) involvement received twice weekly triple intrathecal injections (methotrexate 12.5 mg, hydrocortisone 50 mg, and cytosine 40 mg) till the cerebrospinal fluid cytology was negative along with 24 Gray of cranial radiation therapy. Consolidation therapy consisted of ATO and ATRA for 4 weeks along with two doses of bortezomib at the same dosages as used in induction. Patients who were in molecular remission postconsolidation were offered an auto‐SCT. Patients in molecular remission who were unwilling for an auto‐SCT were offered maintenance therapy with ATO and ATRA given for 10 days in a month for 6 months. These patients also received a dose of bortezomib during each month of maintenance. Post auto‐SCT or during maintenance for those who did not undergo an auto‐SCT, intrathecal methotrexate was administered once a month for 6 months.

### 2.3 Quantification of PML‐RARA copy numbers by RT‐qPCR

The dynamics of molecular remission achieved was evaluated by analyzing the PML‐RARA copy numbers using RT‐qPCR. Peripheral blood was collected from patients every week till the end of induction therapy as described above. Quantification of the *PML‐RARA* transcripts was done using Europe against Cancer (EAC) program protocols.^22^ The RT‐qPCR sensitivity was assessed in‐house using methodology as reported previously.^23^

### 2.4 PML‐RARA sequencing by Illumina RNA fusion kit

Total RNA extracted from malignant promyelocytes was used to selectively enrich for 507 genes that have been reported to be associated with gene fusions in cancer using Illumina Trusight RNA Fusion Panel kit. The RNA was fragmented using divalent cations under high temperature and cDNA was generated from the cleaved RNA fragments using random priming during first and second strand synthesis. Then, sequencing adapters were ligated to the resulting double stranded cDNA fragments. The coding regions of expressed cancer associated genes were captured from this library using sequence specific probes to create the final library. Streptavidin magnetic beads were used to capture probes hybridized to the targeted regions. Two rounds of hybridization ensure the high specificity of the captured regions of interest. The enriched libraries were then sequenced on the Illumina platform Hiseq4000 by 2x100 bp paired‐end sequencing. Bio‐informatics analysis was performed in collaboration with Medgenome Labs Pvt Ltd, Bengaluru, India. The details of bioinformatics analysis are provided in the supplementary methods section.

### 2.5 Outcome variables

The primary outcome measure was safety graded according to the National Cancer Institute Common Terminology Criteria for Adverse Events (NCI CTCAE) version 4.0. The secondary outcome measure was efficacy measured as the proportion of patients achieving molecular remission at the end of induction therapy, event‐free (EFS) and overall survival (OS) at follow‐up. Molecular remission was defined as a negative qualitative RT‐PCR reading with a sensitivity of 10^−4^. For EFS, an event was defined as death or disease relapse at any time after enrolment. For OS, an event was defined as death due to any cause after enrolment.

### 2.6 Statistical analysis and comparison with historical cohort

A comparison was done with an historical cohort of patients who were treated for relapsed APL with a combination therapy similar to the study with the exception of bortezomib. For baseline comparison between groups, Chi‐square test or Fisher's exact test was used for nominal data, Mann‐Whitney U test was used for ordinal and numerical data. For time to event analysis, the comparison of two cohorts was done using the Kaplan‐Meier survival curve with logrank test (unadjusted analysis) for overall survival and event free survival. The variables that were significant at less than 0.05 level in logrank test were considered as potential variables for multivariable Cox‐proportional hazards model (adjusted analysis). The model assumption was evaluated using log‐log (S(t)) vs log time and global test. A value of *P* < .05 was considered as statistically significant. Statistical analysis was performed using SPSS version 21.0.

## 3 RESULTS

### 3.1 Patient characteristics

Between September 2013 and January 2017, 22 patients met the eligibility criteria and were enrolled in this study. The median age was 26.5 years (interquartile range 17.5‐41.5). Fourteen (63.6%) were males. The time from initial diagnosis to relapse was 23.1 months (interquartile range 15.6‐43.8). The *PML‐RARA* transcript was bcr1 in 12 patients (54.5%), bcr2 in 1 patient (4.5%), and bcr3 in 9 patients (40.9%). Fourteen patients (63.6%) were clinically asymptomatic at relapse while others had symptoms like bleeding (four patients), headache (one patient), fever (one patient), and fatigue (two patients). Seven patients (31.8%) had concomitant CNS involvement. The summary of the baseline clinical and laboratory characteristics is provided in Table 1 while the Table S1 provides the data for individual patients.

**Table 1:** Comparison of the clinical and laboratory parameters between the study cohort and the historical cohort

Characteristic	Bortezomib cohort (n = 22) N (%)Mean ± SD/ Median(IQR)	Historical cohort (n = 29) N (%) Mean ± SD/ Median(IQR)	*P* Value
Upfront therapy			
ATO based	21 (95.5)	24 (82.8)	0.218
ATRA based	1 (4.5)	5 (17.2)	
Use of anthracycline in upfront therapy	10 (45.5)	13 (44.8)	0.964
Time from initial diagnosis to relapse (in months)	23.1 (15.6 to 43.8)	20.6 (14.3 to 33.2)	0.481
Age (in years)	26.5 (17.5 to 41.5)	26 (8.0 to 43.0)	0.402
Gender: Male	14 (63.64)	22 (75.9)	0.343
Patients with promyelocytes and blasts in peripheral blood	6 (27.3)	21 (75.0)	**0**.**001**
Hemoglobin (g/dL)	12.7 (10.3 to 13.7)	11.6 (10.2 to 13.6)	0.430
White blood cell count ( in 10^9^/L)	2.65 (1.63 to 6.59)	3.45 (1.43 to 13.13)	0.417
Platelet count ( in 10^9^/L)	112 (37.8 to 154.3)	49 (19.5 to 76.8)	**0**.**010**
Serum creatinine (in mg/dL)	0.75 (0.65 to 0.85)	0.82 (0.64 to 1.00)	0.183
Prothrombin time (in s)	11.8 (11.15 to 13.75)	13.9 (13.0 to 15.5)	**0**.**002**
Activated partial thromboplastin time (in s)	31.7 (28.5 to 32.7)	30.0 (26.1 to 34.8)	0.441
Plasma fibrinogen (in mg%)	200.7 (102.5 to 249.5)	117.45 (82.4 to 158.5)	0.076
Percentage of bone marrow blasts and promyelocytes	64.0 (49.0 to 77.5)	75.5 (67.5 to 90.5)	0.059
Major bleeding at presentation	2 (9.1)	2 (7.4)	1.000
Major thrombosis at presentation	0 (0)	3 (11.5)	0.242
Transfusions during induction			
Packed red cell concentrates	1 (0 to 4)	1.5 ( 0 to 2.3)	0.691
Fresh frozen plasma	0 (0 to 5)	4 (1.5 to 16.3)	**0**.**015**
Cryoprecipitate	0 (0 to 8.5)	5.5 (0 to 9.0)	0.332
Platelet rich concentrate	10 (0 to 29.3)	11 (4.3 to 26.5)	0.690
Patients with molecular remission post induction	19 (90.5)	16 (69.6)	0.137
Duration of follow‐up (in months)	48 (28 to 56.3)	69 (7 to 113.5)	0.361

### 3.2 Treatment response

All patients enrolled in the study achieved hematological remission at the end of induction therapy. The median time to hematological remission was 45 days (range 40‐63). Nineteen patients (90.5%; N = 21. one patient RT‐PCR not sent postinduction) also achieved molecular remission postinduction therapy. All patients achieved molecular remission postconsolidation therapy. Of the 22 patients, 12 opted for autologous transplantation while the rest opted for maintenance therapy. Six patients (27.3%) did not require hospital admission during reinduction therapy. There was no induction death among the patients enrolled in the study.

### 3.3 Safety

Table S2 summarizes the toxicity encountered in the patients on the present study. Grade 3 toxicity was seen in eight instances. Of these, one was motor and sensory neuropathy which required discontinuation of bortezomib. Others were all transient and were clinically managed with the standard of care along with temporary discontinuation of drugs in some cases (1—severe headache resolved after temporary discontinuation of ATRA, 1—eight episodes of loose stools in 1 day postbortezomib which did not recur subsequently, 2—febrile neutropenia requiring hospital admission, 1—oral ulcers causing dysphagia requiring hospital admission, 1—maculopapular rash over involving > 30% of the body surface area which resolved spontaneously, 1—severe backache secondary to bone marrow necrosis which resolved with steroids). Remaining toxicities were grade 1 or 2 and resolved completely with symptomatic management.

### 3.4 Survival analysis

**Figure 2:**
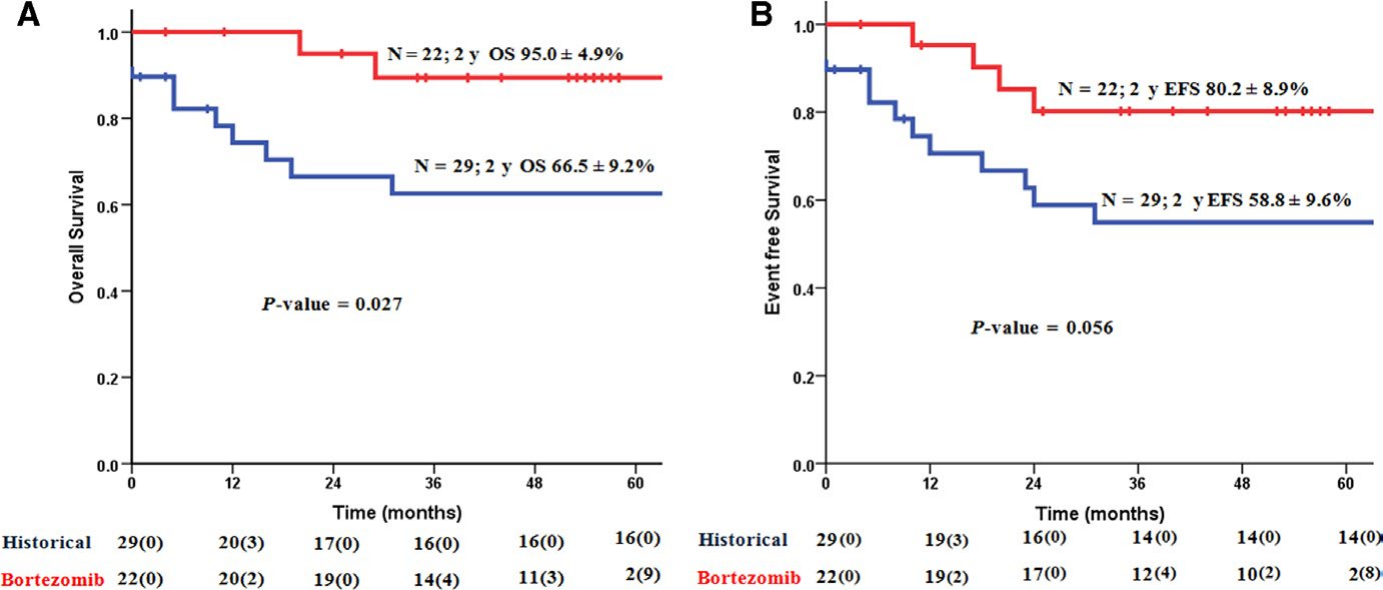
Overall survival (A) and event free survival (B) of the study cohort compared to a historical cohort

The 2‐year overall survival and event‐free survival were 95 ± 4.9% and 80.2 ± 8.9%, respectively (Figure 2A,B). At a median follow‐up of 48 months (range 28‐56.3), 2 (9.1%) patients died (1—disease progression, 1—acute demyelination while in remission) while there were three (13.6%) relapses. All the three patients who relapsed had opted for maintenance therapy postconsolidation. All of them received salvage treatment (retreatment with the induction therapy of the same protocol). Of these, one patient died of disease progression. Of the remaining two patients, one achieved molecular remission after the second salvage treatment while another required additional gemtuzumab ogazamicin to achieve molecular remission. Subsequently both underwent autologous transplantation and are alive at last follow‐up. Figure 2A,B show the overall and event‐free survival of the study cohort alongside that of the historical cohort.

### 3.5 Comparison with the historical cohort

Table 1 and Tables S3‐S5 depict the comparison of the clinical and laboratory characteristics and the treatment outcomes between the study cohort and the historical cohort.

### 3.6 PML‐RARA mutation analysis and dynamics of molecular response

**Figure 3:**
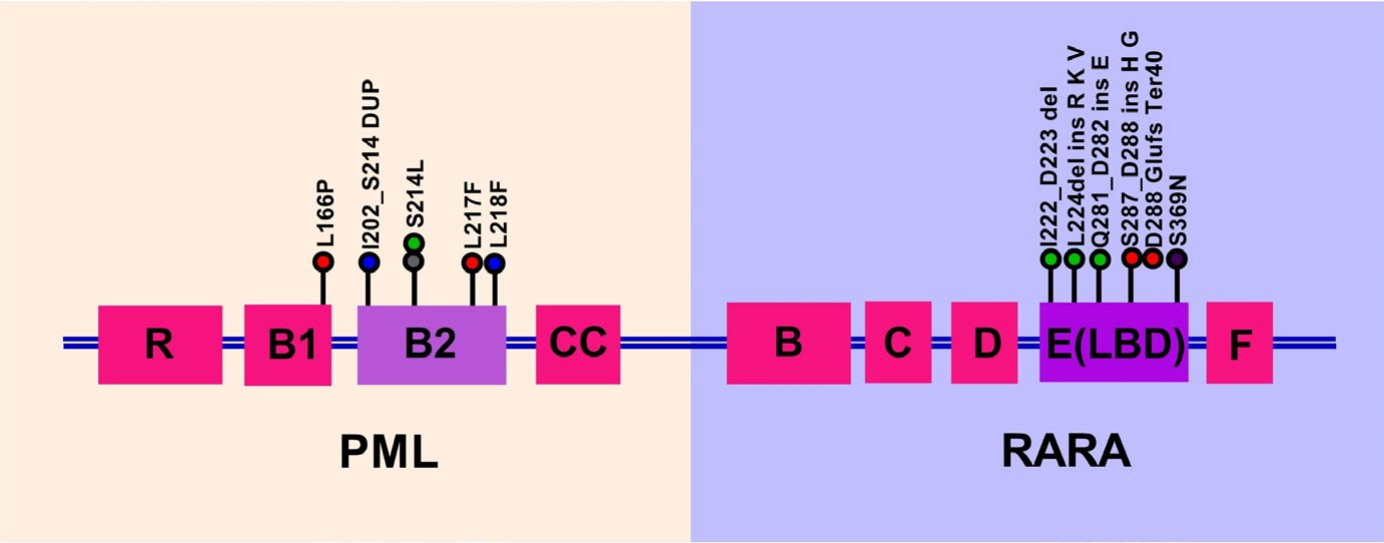
Illustration of mutations identified in PML‐RARA gene using Illumina Trusight RNA Fusion Panel kit. Of the 20 samples analysed, we identified 5 patients carrying mutations in either PML or RARA or both regions. ATO resistance causing mutations such as S214L, L217F and L218F in the B2 domain of PML gene were seen in 4 patients. (Each colour represents one patient)

Out of 20 samples analyzed, we identified eight patients carrying mutations in either PML or RARA or both regions. Figure 3 shows the mutations identified in the PML‐RARA fusion gene in patients in the study cohort. Of these mutations, we observed that the reported ATO resistance causing mutations^19^ in the hot spot of the B2 domain of PML gene such as S214L and L217F as well as a novel L218F mutation (Figure 3) were identified in four patients. Similarly, we noted mutation in ligand binding domain of the RARA gene which may cause resistance to ATRA (Figure 3). However, none of these mutations were associated with secondary ATO resistance in these patients. Based on weekly RT‐qPCR, we noted that most patients achieved complete molecular remission by week 4 of induction therapy as shown in Figure S1.

## 4 DISCUSSION

The standard of care for the treatment of patients with acute promyelocytic leukemia relapsing after frontline treatment with arsenic trioxide is yet to be defined. In patients who have been previously treated with ATO, at relapse, about one‐third harbor mutations in the PML‐RARA fusion gene and the clinical outcomes in this subset are poor.^19^ Besides the mutations in PML‐RARA fusion gene, microenvironment‐mediated drug resistance (EM‐DR) to ATO mediated by the upregulation of the NF‐κB pathway also contributes to drug resistance in relapsed APL.^20^ Recently, in a mouse model experiment, we showed that this EM‐DR could be overcome by proteasome inhibition.^21^

In the present phase II clinical trial, we showed that addition of bortezomib to the combination of ATO, ATRA, and anthracycline is safe. Although there were eight instances of grade 3 adverse events, only one required discontinuation of bortezomib. Thus the treatment regimen was well tolerated with a manageable toxicity profile. We also noted that five patients had mutations in the PML‐RARA fusion gene (Figure 3). Despite this, we noted that with the addition of bortezomib, all patients were in molecular remission post‐consolidation therapy. The RT‐qPCR data (Figure S1) showed that the molecular response was attained by 5th week of induction therapy. As compared to the historical cohort treated without bortezomib, the overall survival of the study cohort was significantly better (Figure 2). All three relapses in the study cohort occurred in patients who did not opt for autologous transplantation. One patient died in remission more than a year after treatment was completed at a secondary hospital and the possibility of an acute demyelinating disease was considered based on the clinical assessment of the treating physician, unfortunately this was not corroborated with a tissue biopsy.

An inherent limitation of the present phase II nonrandomized single arm study was that the historical controls might not have been prognostically comparable to the accrued patients. On comparison of the baseline data of the study cohort with that of the historical cohort, we noted that the historical cohort had greater proportion of patients with peripheral blood blasts or promyelocytes. The patients in the historical cohort also had lower platelet counts and elevated prothrombin time at diagnosis of relapse as compared to the study cohort. Hence they also required more fresh frozen plasma transfusions than the study cohort. Another limitation of the study was that we did not have the mutation and RT‐qPCR data for the historical cohort for comparison.

Despite these limitations, the present study shows that the addition of bortezomib to a combination of ATO, ATRA, and anthracycline is safe and effective. The efficacy needs to be validated in a randomized clinical trial, with an optimized dose and schedule of bortezomib (in the present study dosing of bortezomib was limited to two doses since the main end point was to address safety). If efficacy and safety are proven unequivocally, after optimized dose and scheduling, it may obviate the need for an autologous transplant in second molecular remission. This would be of relevance to patients with relapsed APL especially in developing countries.

## AUTHOR CONTRIBUTION

UK performed research, clinical data accrual, analyzed data, and wrote paper. SG performed research, performed molecular tests, analyzed data, and wrote paper. AAA, HP, SD, NB, and AV performed research, performed molecular tests, flow cytometry tests, and analyzed data. MT and LJ performed statistical analysis. AK, AD, and AA performed research, clinical data accrual, and analysis. NBJ performed research and performed cytogenetic tests. BG performed research, clinical data accrual, and analysis. PB performed research, performed molecular tests, and analyzed data. VM performed research, designed study, clinical data accrual, analyzed data, and wrote paper.
