# Supplementary material for: A phase II study evaluating the role of bortezomib in the management of relapsed acute promyelocytic leukemia treated upfront with arsenic trioxide
Source: Cancer Med. 2020 Feb 14;9(8):2603–10. doi: 10.1002/cam4.2883 (PMC7163093; doi:10.1002/cam4.2883)
Supplement: Supplementary file 1 [file CAM4-9-2603-s001.doc]

**A phase II study evaluating the role of bortezomib in the management of relapsed acute promyelocytic leukemia treated upfront with arsenic trioxide**

Uday Kulkarni^1^, Saravanan Ganesan^1^, Ansu Abu Alex^1^, Hamenth Palani^1^, Sachin David^1^, Nithya Balasundaram^1^, Arvind Venkatraman^1^, , Mani Thenmozhi^2^, Lakshmanan Jeyaseelan^2^, Anu Korula^1^, Anup Devasia^1^, Aby Abraham^1^, Nancy Beryl Janet^1^, Poonkuzhali Balasubramanian^1^, Biju George^1^, Vikram Mathews^1^

1. Department of Haematology, Christian Medical College, Vellore, India.
2. Department of Biostatistics, Christian Medical College, Vellore, India.

**Supplementary:**

**Materials and Methods:**

**1. Detailed Inclusion and Exclusion Criteria:**

**a. Inclusion Criteria:**

i. Diagnosis of relapsed PML-RARα positive APL confirmed by RTPCR.

ii. Normal cardiac function with normal QTc (less than 500 msec) within 48 hours of study entry.

iii. Patient or guardian willing to give informed consent / assent. Must not have a psychiatric disorder(s) that would interfere with consent, study participation, or follow-up.

iv. Patients may have received hydroxyurea, 48 hours or less of ATRA, and 1 dose of an anthracycline and still be eligible for participation in this study.

v. Life expectancy of at least 2 weeks after entry on study.

vi. No age limit for entry into study.

vii. ECOG PS 0, 1, or 2.

viii. Fertile patients must agree to use an effective barrier method of contraception (e.g., latex condom, diaphragm, or cervical cap) to avoid pregnancy while on therapy and for 3 years following the discontinuation of therapy.

ix. Have a negative serum or urine pregnancy test prior to the first dose of therapeutic drugs (if patient is a female of childbearing potential). If breast feeding they should be willing to stop breast feeding.

**b. Exclusion Criteria:**

i. Intracranial bleed at diagnosis.

ii. ECOG performance score 3 and above.

iii. Severe uncontrolled infection, fulminant sepsis at diagnosis or documented pneumonia.

iv. History of cardiac arrhythmia; symptomatic coronary heart disease; uncontrollable arterial hypertension (diastolic blood pressure > 115 mm Hg); severe psychiatric disease or other concomitant diseases which do not comply with the criteria for the participation in the study.

v. Acute hepatitis (Bilirubin≥ 5mg% or liver enzymes ≥ 4 times above laboratory normal value)

vi. Acute renal failure or serum creatinine ≥ 2 mg% not reversed by hydration.

vii. Patients suffering from an additional malignant tumor. No past history of receiving therapy for another malignancy, apart from squamous cell carcinoma or basal cell carcinoma of the skin.

viii. Pregnancy or lactation.

ix. Patients with proven intolerance to the study drugs

x. Inability, missing willingness or anticipated lack of compliance by the principal investigator to participate in the study. Must not have any other severe concurrent disease and/or uncontrolled medical conditions, which, in the judgment of the investigator, could predispose patients to unacceptable safety risks or compromise compliance with the protocol.

1. **Bioinformatics analysis pipeline**

The following bioinformatics steps were performed for analysis of the data

**Read quality check –** It was done using the following parameters from fastq file

- Base quality score distribution
- Sequence quality score distribution
- Average base content per read
- GC distribution in the reads
- Check for over-represented sequences
- Adapter trimming

Based on quality report of fastq files we trim sequence read where necessary to only retain high quality sequence for further analysis. In addition, the low-quality sequence reads are excluded from the analysis. The adapter trimming was performed using fastq-mcf program (version - 1.04.676) and cutadapt version 1.8dev.

**Contamination removal -** Contamination sequence removal was performed using Bowtie2 (version - 2.2.4).

**Read alignment** – The paired-end reads were aligned to the reference human genome Feb. 2009 release downloaded from UCSC database (GRCh37/hg19).The chromosome fasta file was downloaded from the following website (<http://hgdownload.soe.ucsc.edu/goldenPath/hg19/bigZips/chromFa.tar.gz>). GTF file was downloaded from the following website ([ftp://ftp.ensembl.org/pub/release75/gtf/homo_sapiens/Homo_sapiens.GRCh37.75.gtf.gz](ftp://ftp.ensembl.org/pub/release-75/gtf/homo_sapiens/Homo_sapiens.GRCh37.75.gtf.gz)). Alignment was performed using STAR (2.4.1).

**Expression estimation** – The aligned reads are used for estimating expression of the genes and transcripts (or mRNAs) using programs cufflinks (version: cufflinks-2.2.1). The expression values are reported in FPKM (Fragments Per Kilobase of transcript per Million mapped reads) units for each of the genes.

**Identification of Germline variants:** The variant analysis was performed using sentieon GenomeAnalysisTK-3.5 toolkit (HaplotypeCaller).

**Variant annotation** – The identified variants are annotated using our in-house program (VariMAT). The gene model used for annotation was downloaded from Ensembl database Release 84 (<ftp://ftp.ensembl.org/pub/release-75/gtf/homo_sapiens>). The VariMAT performs the following analysis and annotation: Gene and Intergenic annotation, Repeatmasker annotation, Exon, intron, 5UTR, 3UTR, coding-region, splice-site annotation, Mapping of variants to all transcript form of the gene, Variant class prediction – silent, missense, non-sense, stop-loss, start-loss, in-frame, frameshift. dbSNP, 1000genome, HapMap, Comparison with ClinVar, HPMD, GWAS, COSMIC, OncoMD, PolyPhen, SIFT, MutationTaster, MutationAssessor, LRT and others.

**Identification of fusions:** Fusion detection was performed using NCLscan (V1·6: <https://github.com/TreesLab/NCLscan>) and FusionCatcher (V0·99·7c: <https://github.com/ndaniel/fusioncatcher>).

**Visualization of gene fusion:** AGFusion (V0·134: (<https://github.com/murphycj/AGFusion>) was used to visualize gene fusion and annotations.

**Bioinformatics analysis pipeline:**

**
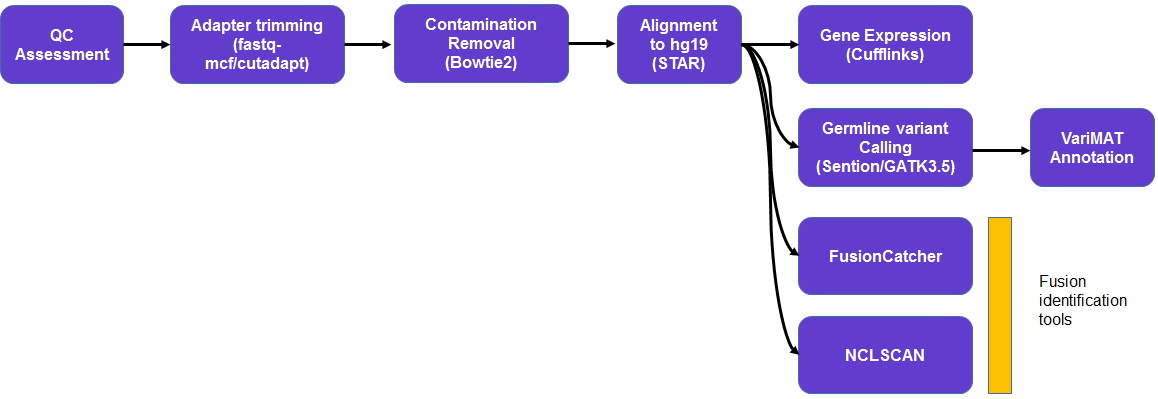
**

Figure 1: Bioinformatics analysis pipeline

**
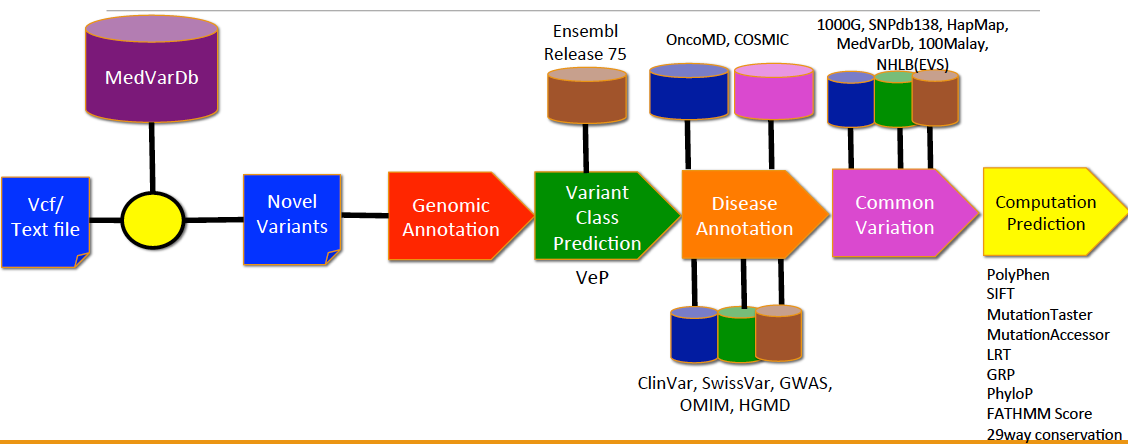
**

Figure 2: Variant annotation pipeline

**Supplementary Figure 1: Dynamics of the molecular remission achieved in relapsed patients enrolled (n=19; available samples) in this study** (NCN- Normalized copy number; normalized to ABL transcripts; PML-RARA(NCN) = copy number of PML-RARA x 100/copy number of ABL). All patients attained complete molecular remission by week 5.

**
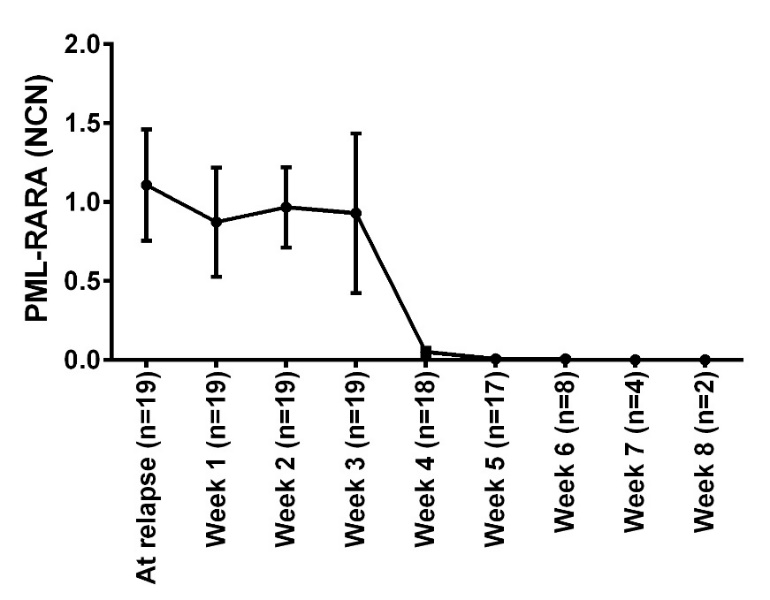
**

**Supplementary Table 1:** please see separate excel sheet.

**Supplementary Table 2:** Toxicity encountered in the patients enrolled in the study protocol as per NCI CTCAE version 4.

| **Toxicity** | **Grade 1** | **Grade 2** | **Grade 3** |
| --- | --- | --- | --- |
| Febrile neutropenia | 0 | 0 | 2 |
| Maculopapular rash | 0 | 2 | 1 |
| Headache | 7 | 1 | 1 |
| Diarrhea | 1 | 1 | 1 |
| Neuropathy | 1 | 0 | 1 |
| Backache | 1 | 0 | 1 |
| Dysphagia | 0 | 0 | 1 |
| Depressed level of consciousness | 0 | 1 | 0 |
| Muscle weakness | 0 | 1 | 0 |
| Uveitis | 0 | 1 | 0 |
| Vomiting | 1 | 0 | 0 |
| Infections involving the skin | 1 | 0 | 0 |
| Impaired hearing | 1 | 0 | 0 |
| Cough | 1 | 0 | 0 |
| Vertigo | 1 | 0 | 0 |
| Blurred vision | 1 | 0 | 0 |
| Sore throat | 1 | 0 | 0 |
| Involuntary movements | 1 | 0 | 0 |
| **Total** | **18** | **7** | **8** |

**Supplementary Table 3: Unadjusted Cox proportional hazard model – Overall survival**

| **Variable** | **HR** | **95% - CI** | **p value** |
| --- | --- | --- | --- |
| **Group**  Historical cohort  Bortezomib | 4·69  1·00 | 1·03 – 21·48 | **0·046** |
| **Sex**  Male  Female | 1·00  0·70 | 0·19 – 2·58 | 0·589 |
| **Age** | 1·02 | 0·98 – 1·06 | 0·393 |
| **Hemoglobin** | 0·90 | 0·72 – 1·13 | 0·353 |
| **Total WBC count** | 1·01 | 1·00 – 1·02 | **0·022** |
| **Platelet count** | 1·00 | 0·99 – 1·01 | 0·397 |
| **Serum creatinine** | 1·92 | 0·13 – 29·23 | 0·637 |
| **Prothrombin time** | 1·15 | 0·92 – 1·44 | 0·209 |
| **Activated partial thromboplastin time** | 0·97 | 0·87 – 1·08 | 0·594 |
| **Fibrinogen** | 1·00 | 0·99 – 1·01 | 0·961 |
| **Bone marrow blasts + promyelocytes** | 1·02 | 0·99 – 1·06 | 0·199 |
| **Major bleeding**  Yes  No | 1·16  1·00 | 0·15 – 9·06 | 0·888 |
| **Major thrombosis**  Yes  No | 1·72  1·00 | 0·22 – 13·49 | 0·603 |
| **Fresh frozen plasma transfusions** | 1·03 | 0·98 – 1·08 | 0·320 |
| **Cryoprecipitate transfusions** | 0·91 | 0·79 – 1·04 | 0·161 |
| **Platelet transfusions** | 1·01 | 0·97 – 1·04 | 0·729 |
| **Packed red cell concentrate transfusions** | 1·09 | 0·82 – 1·45 | 0·536 |
| **Autologous transplantation**  Yes  No | 0·29  1·00 | 0·07 – 1·19 | 0·087 |
| **RTPCR end induction**  Positive  Negative | 1·23  1·00 | 1·23 – 0·26 | 0·796 |

**Supplementary T**able 4: Unadjusted Cox proportional hazard model – Event free survival

| **Variable** | **HR** | **95% - CI** | **p value** |
| --- | --- | --- | --- |
| **Group**  Historical cohort  Bortezomib | 2·85  1·00 | 0·92 – 8·85 | 0·070 |
| **Sex**  Male  Female | 1·00  0·72 | 0·23 – 2·23 | 0·568 |
| **Age** | 1·01 | 0·97 – 1·04 | 0·769 |
| **Hemoglobin** | 0·93 | 0·77 – 1·14 | 0·501 |
| **Total WBC count** | 1·01 | 1·00 – 1·02 | **0·004** |
| **Platelet count** | 1·00 | 0·99 – 1·01 | 0·539 |
| **Creatinine** | 1·42 | 0·13 – 15·08 | 0·774 |
| **Prothrombin time** | 1·26 | 1·05 – 1·51 | **0·012** |
| **Activated partial thromboplastin time** | 0·99 | 0·92 – 1·06 | 0·678 |
| **Fibrinogen** | 1·00 | 0·99 – 1·01 | 0·985 |
| **Bone marrow blasts + promyelocytes** | 1·02 | 0·99 – 1·04 | 0·226 |
| **Major bleed**  Yes  No | 2·10  1·00 | 0·47 – 9·34 | 0·328 |
| **Major thrombosis**  Yes  No | 1·20  1·00 | 0·16 – 9·15 | 0·859 |
| **Fresh frozen plasma transfusions** | 1·06 | 1·02 – 1·10 | **0·005** |
| **Cryoprecipitate transfusions** | 0·96 | 0·90 – 1·03 | 0·258 |
| **Platelet transfusions** | 1·02 | 0·99 – 1·06 | 0·115 |
| **Packed red cell concentrate transfusions** | 1·13 | 0·89 – 1·42 | 0·311 |
| **Autologous transplantation**  Yes  No | 0·26  1·00 | 0·08 – 0·82 | **0·021** |
| **RTPCR end induction**  Positive  Negative | 0·91  1·00 | 0·20 – 4·20 | 0·901 |

**Supplementary Table 5**: Multivariate analysis for overall survival

| **Variable** | **HR** | **95% - CI** | **p value** |
| --- | --- | --- | --- |
| **Group**  Historical cohort  Bortezomib | 22·65  1·00 | 1·59 – 323·69 | **0·021** |
| **Total WBC count at relapse** | 1·00 | 0·97 – 1·04 | 0·789 |
| **Autologous transplantation**  Yes  No | 0·02  1·00 | 0·001 – 0·28 | **0·004** |
